# Site-Specific Mobilization of Vinyl Chloride Respiration Islands by a Mechanism Common in *Dehalococcoides*

**DOI:** 10.1186/1471-2164-12-287

**Published:** 2011-06-02

**Authors:** Paul J McMurdie, Laura A Hug, Elizabeth A Edwards, Susan Holmes, Alfred M Spormann

**Affiliations:** 1 Department of Civil and Environmental Engineering, Stanford University, Stanford, California, USA; 2 Department of Cell and Systems Biology, University of Toronto, Toronto, Ontario, Canada; 3 Department of Chemical Engineering and Applied Chemistry, University of Toronto, Toronto, Ontario, Canada; 4 Department of Statistics, Stanford University, Stanford, California, USA; 5 Department of Chemical Engineering, Stanford University, Stanford, California, USA

## Abstract

**Background:**

Vinyl chloride is a widespread groundwater pollutant and Group 1 carcinogen. A previous comparative genomic analysis revealed that the vinyl chloride reductase operon, *vcrABC*, of *Dehalococcoides *sp. strain VS is embedded in a horizontally-acquired genomic island that integrated at the single-copy tmRNA gene, *ssrA*.

**Results:**

We targeted conserved positions in available genomic islands to amplify and sequence four additional *vcrABC *-containing genomic islands from previously-unsequenced vinyl chloride respiring *Dehalococcoides *enrichments. We identified a total of 31 *ssrA*-specific genomic islands from *Dehalococcoides *genomic data, accounting for 47 reductive dehalogenase homologous genes and many other non-core genes. Sixteen of these genomic islands contain a syntenic module of integration-associated genes located adjacent to the predicted site of integration, and among these islands, eight contain *vcrABC *as genetic 'cargo'. These eight *vcrABC *-containing genomic islands are syntenic across their ~12 kbp length, but have two phylogenetically discordant segments that unambiguously differentiate the integration module from the *vcrABC *cargo. Using available *Dehalococcoides *phylogenomic data we estimate that these *ssrA*-specific genomic islands are at least as old as the *Dehalococcoides *group itself, which in turn is much older than human civilization.

**Conclusions:**

The *vcrABC *-containing genomic islands are a recently-acquired subset of a diverse collection of *ssrA*-specific mobile elements that are a major contributor to strain-level diversity in *Dehalococcoides*, and may have been throughout its evolution. The high similarity between *vcrABC *sequences is quantitatively consistent with recent horizontal acquisition driven by ~100 years of industrial pollution with chlorinated ethenes.

## Background

Chlorinated ethene congeners ("chloroethenes") are among the most frequently detected groundwater contaminants in the United States of America and other industrialized countries [1]. Chloroethenes are often incompletely dechlorinated by bacteria in these anoxic environments, leading to an accumulation of vinyl chloride, a Group 1 human carcinogen [2,3]. Growth-linked reductive dechlorination of vinyl chloride is critical to avoid its accumulation and achieve *in situ *remediation of chloroethenes [1], but vinyl chloride respiration has only been observed in certain strains of *Dehalococcoides *[4,5]. *Dehalococcoides *is a genus-level phylogenetic group within the *Chlorofiexi *phylum [6]. *Dehalococcoides *are strictly anaerobic bacteria that gain metabolic energy exclusively via the oxidation of H_2 _coupled to the reduction of organohalide compounds [7-9]. This catabolic reductive dehalogenation of organohalide compounds ("organohalide respiration") is catalyzed in *Dehalococcoides *by heterodimeric, membrane-bound enzymes called "reductive dehalogenases" [10]. Reductive dehalogenases typically contain corrinoid and iron-sulfur clusters as cofactors, and have varied substrate ranges that do not necessarily overlap [10,11].

The catalytic subunit of reductive dehalogenases is encoded in *Dehalococcoides *by reductive dehalogenase homologous genes (*rdhA*). *Dehalococcoides *possess as many as 36 *rdhA *per genome [9], but few of the encoded enzymes, RdhA, have been purified and characterized *in vitro*. Many *rdhA *are co-expressed [12-16], further confounding a determination of the RdhA responsible for catalysis of an observed reductive dehalogenation activity. The only reductive dehalogenase shown to catabolically reduce vinyl chloride, VcrA, was purified from a highly-enriched vinyl chloride respiring culture dominated by *Dehalococcoides *strain VS [5]. The operon encoding VcrA, *vcrABC*, was identified by reverse genetics, and highly-similar *vcrA *were detected in other vinyl chloride respiring *Dehalococcoides *cultures [5,17,18]. Primers targeting *vcrA *are now commonly used as an indicator of attenuation potential at vinyl chloride contaminated sites ([5], U.S. Patent Application 20090176210). A putative VC reductase operon, *bvcAB*, shares only limited similarity with *vcrAB *and is present in a different VC respiring *Dehalococcoides *strain, BAV1, which does not contain *vcrABC *[19].

Although *Dehalococcoides *are the only known microorganisms capable of vinyl chloride respiration, both *vcrA *and *bvcA *appear to be horizontally acquired [9]. Both *vcrA *and *bvcA *have a highly unusual, low %(G+C) codon bias that appears maladapted to *Dehalococcoides *genomes [20], and both are found within a low %(G+C) "genomic island" (GI) [21] that interrupts local gene synteny relative to other *Dehalococcoides *strains. In strain VS, this *vcrABC *-containing genomic island (*vcr*-GI) integrated at the *ssrA *locus, and as a result is flanked by *ssrA *and a 20 bp direct repeat of the *ssrA *3' end [9]. *ssrA *is a single-copy gene essential in bacteria [22] encoding transfer messenger RNA (tmRNA), which plays a key role in maintaining the fidelity of protein synthesis [23]. Specific integration of genetic elements at *ssrA *is also common across many bacterial phyla, and often results in a direct repeat at the genomic island boundary opposite the site of integration [24]. In addition to the *vcr*-GI, over a dozen *ssrA *direct repeats were previously detected downstream of *ssrA *in *Dehalococcoides*, collocated with many strain-specific *rdhA *in a region of high genomic variability between *Dehalococcoides *strains [9]. To further understand the acquisition and dissemination of *vcrABC*, as well as the impact of *ssrA*-specific integration on *Dehalococcoides *genome dynamics, we determined the conserved features of *Dehalococcoides ssrA*-specific genomic islands (*ssrA*-GIs) from all publicly available genomes and metagenomes of *Dehalococcoides *cultures, including the recently-sequenced *Dehalococcoides *strain GT [17] and the metagenome sequences of the vinyl chloride respiring *Dehalococcoides *enrichment cultures KB-1 [25,26] and ANAS [27]. We also amplified and sequenced *ssrA*-GIs from the vinyl chloride respiring *Dehalococcoides *enrichment cultures Evanite (EV) [28], PM [28], WBC-2 [29], and WL [30] using primers designed to target either *vcr*-GIs specifically, or conserved features present in all available *Dehalococcoides ssrA*-GIs. Previous studies have implicated a subset of *rdhA*B with horizontal gene acquisition, but evidence for the method of integration, mobilization, replication, and transfer is limited [31,32]. We describe here a family of putative *ssrA*-specific integrative and mobilizable elements [33] that share a conserved 'integration module' while also encoding a broad variety of putative and unknown functions, including reductive dehalogenation. The key conserved integrase encoded on these elements is a homolog of the CcrB family of site-specific serine recombinases that specifically integrate/excise the methicillin-resistance element "SCCmec" in *Staphylococcus aureus *[34]. Using a robust whole-genome phylogeny and several estimates for mutation rate, we estimate the age of the most recent common ancestor of contemporary *Dehalococcoides *strains, as well as the age of divergence for *Dehalococcoides ssrA*-GI integration module components.

## Results

### *ssrA *Genomic Islands in *Dehalococcoides*

**Figure 1:**
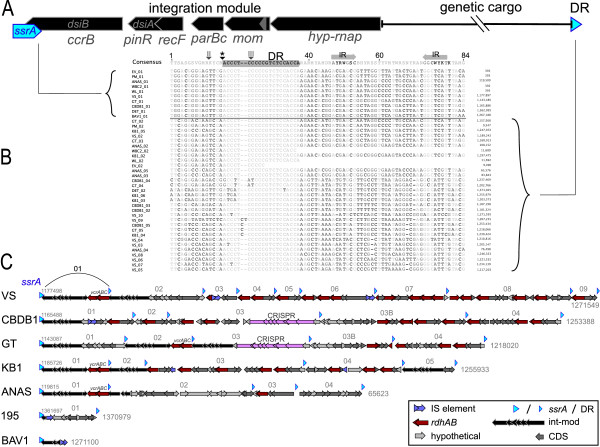
**General structure of *ssrA *genomic islands (*ssrA*-GIs)**. (**A**) Generalized structure of *Dehalococcoides ssrA*-GIs, oriented according to published *Dehalococcoides *complete genomes. Labels below genes in the integration module indicate the most informative homolog of the protein encoding gene. (**B**) Alignment of the 84 bp region surrounding the 3' end of *ssrA *or its direct repeat (DR) fragments (shaded black in the consensus) from 40 such positions in available *Dehalococcoides *genomes. Each sequence is labeled by its strain or enrichment name, underscore, and the order in which it occurs, beginning with the 3' end of *ssrA*. Positions in the alignment that disagree with the 75% consensus sequence are shaded in darker grey. The alignment is ordered such that sequences corresponding to *Dehalococcoides ssrA *("_01") are the top 10 sequences, emphasizing a conserved position of disagreement between *ssrA *sequences and the direct repeat regions, position 15 in the alignment, 333 in *ssrA*. The two bases flanking the inferred integration site are marked with a hash. (**C**) To-scale genomic maps of region downstream of *Dehalococcoides ssrA *in (meta)genomic datasets. Orientation of genes is indicated with arrows. Key genes are shaded according to the provided legend.

The region downstream of *ssrA *in available *Dehalococcoides *(meta)genome sequences contains multiple tandem genomic islands that are primarily distinguished by their boundaries - *ssrA *or its 20 bp direct repeat - as well as disruption to local gene synteny and in many cases the presence of a characteristic cluster of integration-associated genes adjacent to the left edge (Figure 1). All direct repeats are located within 100 kbp downstream of *ssrA*, with varying numbers per strain and no duplicate genomic islands within any strain. These findings are consistent with *ssrA*-specific integration described for other bacteria [21,24], as well as a class of integrating and mobilizing elements that encode their own specific integration but do not replicate independently from the chromosome nor encode for conjugation [33]. From available *Dehalococcoides *genomic data (including this study) we have detected a total of 31 *ssrA*-GIs containing 47 *rdhA*, 75 hypothetical protein encoding genes, 2 putative complete CRISPR modules and arrays [35], as well as other genes; most of which are not believed to encode a core function and are present in only a subset of *Dehalococcoides *strains.

Sixteen of the identified *Dehalococcoides ssrA*-GIs contain an integration module comprised of 6 syntenic protein encoding genes oriented on the reverse strand and located adjacent to *attL *(in this context, *attL *and *attR *are the *ssrA *direct repeat sequence at the left or right boundary, respectively; Figure 1A).

**Figure 2:**
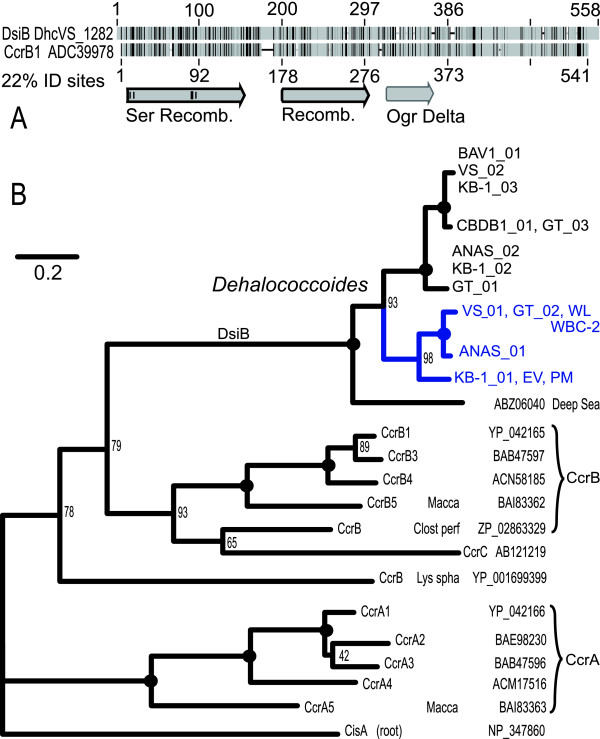
**Phylogeny of *ssrA*-GI integrase, DsiB**. (**A**) Grey-scale similarity 'barcode' representation (black is identical sites) of pairwise global alignment (Needleman-Wunsch, free end-gaps) between a representative DsiB [DhcVS_1282, Genbank: ACZ62382], and CcrB1 of *Staphylococcus aureus *[Genbank: ADC39978]. Key domains of CcrB1 are annotated below the alignment, and traced in black if they are also detected in DhcVS_1282 by the conserved domain database search [84] incorporated in PSI-BLAST [85]. (**B**) Maximum Likelihood tree of the putative integrases encoded on *Dehalococcoides ssrA*-GIs, DsiB, as well as key integrases involved in mobility of SCCmec in *Staphylococcus aureus *[64] (unless otherwise noted). The clade of integrase sequences found on *vcr-*GIs are shaded in blue. Nodes with 100% bootstrap support are bolded with a filled circle. CisA of *Clostridium acetobutylicum *ATCC 824 is rooted as an outgroup, as in [86]. The following abbreviations are used to label CcrA, CcrB, or CcrC from bacteria other than *S. aureus*: 'Lys spha' - *Lysinibacillus sphaericus *C3-41; 'Clost perf' - *Clostridium perfringens *C str. JGS1495; 'Macca' - *Macrococcus caseolyticus *[86].

Beginning from *attL*, the integration module contains genes that appear to encode (1) a 540 residue serine recombinase family putative site-specific integrase we call *Dehalococcoides ssrA*-specific integrase, DsiB (Figure 2); (2) a smaller (200aa) PinR (COG1961) homolog that also contains a serine recombinase catalytic domain (cd00338), DsiA; (3) a small (150aa) RecF homolog likely involved in DNA recombination or repair [36], (4) a 210 residue protein with ParBc domain, possibly catalyzing single-stranded DNA cleavage, circular element nicking, element segregation ([37,38], PF02195); (5) a Mom [39] homolog (270 aa), predicted to play a role in restriction endonuclease resistance via methylation [40,41]; and (6) a large (700 aa) protein containing a DNA-directed RNA polymerase domain in the first 85 residues (GO:0003899). These integration modules also contain a 76 bp conserved tRNA-like locus embedded within the first 150 bp of the fifth protein encoding gene, approximately 4400 bp from *attL *(Figure 1A). It is usually labeled as 'pseudo-tRNA' by automated annotation pipelines, but alignment-based RNA folding analysis predicts a complete tRNA-Gly-like structure (Additional file 1 Figure S1). The elevated sequence conservation at its 3' end provides an effective target for primers, as do the regions surrounding *ssrA *direct repeats and a site of locally high nucleotide conservation within *dsiB *(Figures 1B, Additional file 1 Figure S2).

Alignment of the ~85 bp surrounding each of the 28 *ssrA *direct repeats reveals additional nucleotide conservation and allows identification of the site of insertion in *ssrA *(Figure 1B). A 15 bp motif (TTCAGRSMGMRKCCA) occurs adjacent upstream of the direct repeat and does not align well with the corresponding positions in *Dehalococcoides ssrA *(318-333), indicating that insertion likely occurs between 333 and 334 in *ssrA*. This location corresponds to the middle of the T-loop of the encoded tmRNA, between the canonical insertion positions called 'Sublocations II and III' [24] (Figure 1B).

### Specific features of *vcr*-GIs

**Figure 3:**
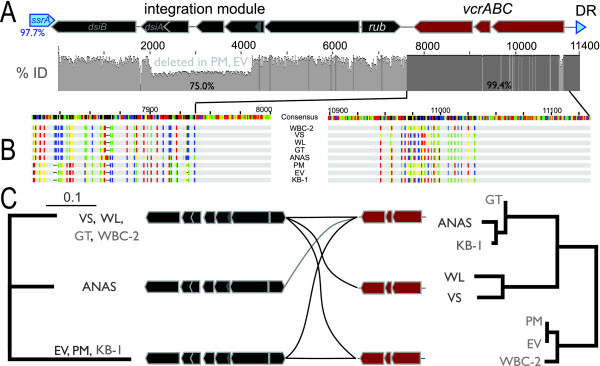
**Organization, alignment, and phylogenetic comparison of 8 vinyl chloride reductase genomic islands (*vcr-*GIs)**. (**A**) To scale summary plot (0 - 100% ID. 14 bp window) of a multiple alignment of all 8 *vcr-*GIs. Horizontal axis numbers indicate the distance downstream of *ssrA*, in nucleotides. Bar heights are shaded darker grey when their value is 100%. Position and orientation of genes are annotated above the plot, shaded according to Figure 1. Regions of categorically different similarity correspond to the integration and *vcrABC *cargo modules, with region-wide average % IDs of 75.0 and 99.4, respectively. (**B**) Enlarged view of the multiple alignment at key positions. (Left) The presumed boundary between integration and cargo modules. (Right) The region of atypically high substitutions occurring in the leader sequence of *vcrA*. Identical sequence is shaded light-grey, nucleotides that disagree with the consensus are indicated with tick marks shaded red, green, yellow or blue representing nucleotides A, T, G, C, respectively. (**C**) Phylogenetic discontinuity between integration modules (left) and their attached *vcrABC *cargo (right), represented by separately calculated Maximum Likelihood trees. Middle cartoon summarizes the major phylogenetic separations of the trees, with curves connecting modules if one of the 8 *vcr-*GIs contains the corresponding combination of module types. *vcr-*GIs sequences are from *Dehalococcoides *strain VS [GenBank:CP001827], strain GT [GenBank:NC_013890]) and two metagenomes (KB-1 [JGI:4083612], ANAS [JGI:4085297]), as well as targeted sequencing (this study) from the vinyl chloride-respiring *Dehalococcoides *enrichment cultures WBC-2, PM, EV, and WL [GenBank:JN034252-JN034255].

*vcr*-GIs are a distinct subset of *Dehalococcoides ssrA*-specific genomic islands, present in two *Dehalococcoides *genomes (VS [GenBank:CP001827], GT [GenBank:NC_013890]) and two metagenomes (KB-1 [JGI:4083612], ANAS [JGI:4085297]). Using primers that target conserved features of all *ssrA*-GIs or specific features of *vcrABC*, we amplified and sequenced 4 additional *vcr*-GIs from independently derived vinyl chloride respiring *Dehalococcoides *enrichment cultures (WBC-2, PM, EV, WL [GenBank:JN034252-JN034255] see Methods). In all instances the *vcr*-GI is located immediately adjacent to *ssrA*, except strain GT where it is the second genomic island downstream of *ssrA*. Because *ssrA *is an essential single-copy gene encoding a structural RNA [42], its sequence provides a coarse phylogenetic identity of the chromosome from which it was amplified [43]. This allowed confirmation that the *vcr*-GIs acquired via metagenomic and targeted sequencing are *Dehalococcoides *chromosomal segments, even though the source genomic DNA was from a mixed culture (Figure 3, Additional file 1 Figure S3).

These *vcr*-GIs contain integration and cargo (*vcrABC *) modules with discordant evolutionary histories. The boundary between integration module and *vcrABC *is delineated by an unambiguous difference in nucleotide identity, 75.0 and 99.4%, respectively (Figure 3). This boundary reveals that *vcr*-GI integration modules contain a seventh protein encoding gene oriented in the opposite (forward) direction encoding a rubredoxin domain protein, in addition to the 6 integration module genes described previously (Figure 3). These integration modules (9164 - 11361 bp) are related as three distinct branches with nearly-identical leaves (masking a ~2200 bp deletion in PM, EV), grouped in a topology that is discordant with the corresponding tree of the 3784 bp *vcrABC *cargo (Figure 3). Relatedness of *vcrABC *-cargo sequences was estimated based on the 44 variant positions in their alignment, appearing mostly (66%) in the form of substitutions in the leader sequence of *vcrA *[5] (Figure 3). *K_a_*/*K_s _*ratios for the *vcrA *leader sequence (first 129 bp) ranged between approximately 0.05 and 0.2 for different pairwise combinations [44] and phylogenetic nodes [45], suggesting this region is under purifying selection. By contrast, the *K_a_*/*K_s _*ratio for the remainder of *vcrA *was incalculable because all 15 variant positions (out of 1431 bp) were non-synonymous substitutions, suggesting recent positive selection on the mature *VcrA *enzyme. This latter conclusion must be tempered by the limited information available in just 15 variant sites, the complete lack of indels detected in any *vcrA *(suggesting some purifying selection in the leader sequence), as well as the clear influences of recent horizontal gene transfer and recombination on these *vcr*-GIs. For example, *vcrC *is identical across all strains, within a 1650 bp region of perfect identity.

### Age of *Dehalococcoides*

A core-gene phylogenetic tree was constructed to support age estimates based on evolutionary models. The core-gene tree was built from 432 core orthologous protein encoding genes shared between available *Dehalococcoides *(meta)genomes and *Dehalogenimonas lykanthroporepellens *BL-DC-9, a *Chlorofiexi *strain that is a phylogenetic outgroup to *Dehalococcoides *and its closest completely-sequenced relative [46]. Age estimates depend heavily on the assumed rate of mutation. We iterated our calculations on multiple published mutation rates (see Methods), as well as an empirical observation for mutation rate derived from the known divergence time (16 years, S. Zinder, pers. comm.) between the isolation of *Dehalococcoides ethenogenes *strain 195 [6] and the generation of a metagenome of its parent culture, DONNA2 (R. E. Richardson, pers. comm.). The latter empirical rate is substantially faster than the published values of faster-growing microbes (Additional file 4 Table S1), possibly because it includes mutations that already existed between strain variants within the DONNA2 culture prior to isolation of strain 195. Although we expect a long-term average mutation rate in the natural environment to be slower, and hence ages based on this rate to be an underestimate, it remains useful as a conservative bound on the 'recentness' of the events in question. Similarly, we used a range of growth rates to estimate the age of *Dehalococcoides*. For a recent bound we used the fastest reported *Dehalococcoides *doubling time (0.8 days [6]), as well as a range of slower reported growth rates from anaerobic environmental systems for more realistic estimates (11-14 days [47-49]). The corresponding estimates and lower (recent) bounds are presented in Table 1.

**Table 1:** Divergence Time Estimates Under Different Rates of Evolution.

Divergence of interest	Tree Calculation Method	Divergence time estimates from different proposed rates					
		**Universal bacterial rate** **in nature**		**Empirical *E. coli *rates** **in culture**		**DONNA2/** **strain 195** **divergence**	**16S clock**
		
*Dehalogenimonas/Dehalococcoides *MRCA	Splitstree	5	(1.9/28/33/34)	0.9	(0.4/5.3/6.2/6.3)	0.5	200-600
	ML	3	(1.2/18/21/21)	0.5	(0.2/3.2/3.8/3.9)	0.3	
*Dehalococcoides *MRCA	Splitstree	0.3	(0.14/2/2.4/2.4)	0.06	(0.03/0.37/0.44/0.44)	0.04	30-60
	ML	0.4	(0.17/2.5/3/3)	0.08	(0.03/0.47/0.55/0.56)	0.04	
*ssrA*-GI integration modules MRCA	ML	3	(1.1/16/19/19)	0.5	(0.2/3/3.5/3.6)	0.2	
integration modules MRCA, *vcr*-GIs only	ML	1	(0.4/5.5/6.5/6.6)	0.2	(0.07/1/1.2/1.2)	0.08	
*vcrAB *MRCA	ML	0.05	(0.02/0.27/0.31/0.32)	0.008	(0.03/0.47/0.55/0.56)	0.004	
*vcrAB*, leader masked	ML	0.01	(0.004/0.057/0.067/0.068)	0.002	(0.001/0.010/0.012/0.013)	0.0009	

Reported age estimates (left, no parenthesis) are based on the rate of evolution listed in the column header as well as the average *Dehalococcoides *doubling time from published values (2 days). All estimates are reported in units of 1 million years. Age estimates of the *Dehalococcoides *clade from the Splitstree consensus network are based on branch lengths of strains to the common network node. '16S clock' is based on 1-2% 16S rRNA gene divergence per 50 million years [83]. Values in parenthesis are results from different doubling time estimates: 0.8 days - the fastest reported doubling time for *Dehalococcoides *[6]; 11.71 days - anaerobic benthic sediment community doubling time [49]; 13.76 days - bacterial doubling time in anaerobic seawater [48]; 14 days - approximate doubling time of the strictly anaerobic annamox bacteria [47]. For the secondary calculations of age estimates, an extra significant digit has been reported to allow distinguishing of estimates.

**Figure 4:**
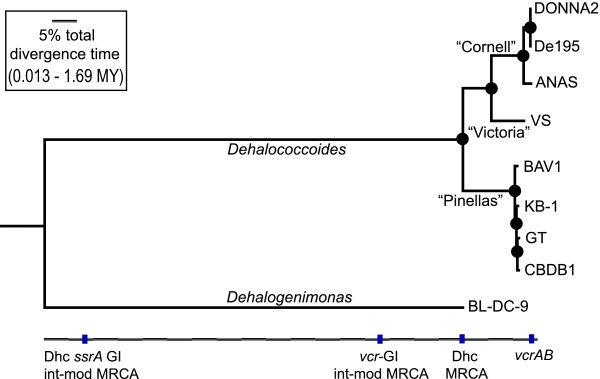
**Date Estimates of Key Events in *Dehalococcoides *Evolution**. Maximum likelihood phylogeny of 432 'core' orthologs. Timing of key evolutionary events are mapped onto the tree. The horizontal line below the tree represents the divergence time to the *Dehalococcoides *(Dhc) and *Dehalogenimonas *(Dehly) MRCA, while vertical hash marks indicate the relative divergence times of *Dehalococcoides ssrA *genomic island (GI) components. These include *Dehalococcoides ssrA *integration modules, *vcrABC*-attached integration modules, and *vcrAB*. Relative divergence times are based on the estimated age of the MRCA of *Dehalococcoides *and *Dehalogenimonas *(set to 1). The scale bar represents 5% of the total divergence time. Absolute time scales are from published mutation rate estimates and rate estimates based on the *Dehalococcoides *strains DONNA2/strain 195 divergence. Black points on the tree are nodes with 100% bootstrap support.

In relative terms, the divergence of *Dehalococcoides *and *Dehalogenimonas *are comparable to the predicted most recent common ancestor (MRCA) of available integration modules, approximately an order of magnitude earlier than the MRCA of *Dehalococcoides *strains. The MRCA of *vcr*-GI integration modules also significantly precedes the divergence of contemporary *Dehalococcoides *strains. In contrast, the high similarity among *vcrABC *sequences results in an estimated age that is at least an order of magnitude younger than *Dehalococcoides *speciation (Figure 4, Table 1).

## Discussion

### Age and specific features of *vcrABC *acquisition

Nucleotide similarity is significantly higher between *vcrABC *cargo modules than can be expected if it was an orthologous locus present in the *Dehalococcoides *common ancestor (Figures 3, 4). In all cases *vcrABC *is located within a syntenic putatively-mobile element, *vcr-*GI, that is part of a broader class of *ssrA*-specific mobile elements that appear to be common among *Dehalococcoides*. In all *vcrABC *-containing strains except GT, the *vcr-*GI is located adjacent to the primary site of integration, *ssrA*, structural evidence that *vcr-*GIs are among the most recently integrated of the available *Dehalococcoides ssrA*-GIs. Within phylogenetic branches, integration modules are perfectly identical, except for a large identical deletion in the EV and PM *vcr-*GIs. The significantly unusual nucleotide signature of *vcr-*GIs [9,20], as well as the discordance between the *vcrA *tree and the corresponding *Dehalococcoides *strain phylogeny, indicate that *vcrABC *has not been stably maintained in *Dehalococcoides *genomes since their divergence. Taken together, these observations suggest recent horizontal acquisition and dissemination of *vcrABC *across all *Dehalococcoides *ecotypes by way of a *ssrA*-specific mobile element with conserved attachment site and integration module.

Because anthropogenic release of chloroethenes into the environment is a relatively recent phenomenon (~100 years [3]), we are particularly interested in the recent bounds for estimates of the age of the MRCA of these *vcrABC *sequences as a proxy for their horizontal acquisition by *Dehalococcoides*. Using our highest estimated rates of mutation and chromosomal replication, the divergence of these *vcrABC *sequences appears to have occurred 4000 years ago. This value is in flated by the inexplicably high variation within the leader sequence of *vcrA*. If we remove the *vcrA *leader sequence from the calculation, the age of divergence decreases to 900 years. However, there is clear signal for positive selection in the remaining *vcrAB *sequence alignment: all 16 variant positions (15 in *vcrA *and 1 in *vcrB*) are predicted to result in amino acid substitutions. If positively selected, these mutations may have accumulated faster than the background rates assumed in our molecular dating calculations. Because the relative increase in substitution rate is unclear and the total information represented by just 16 variant positions is low, we cannot confidently distinguish the divergence of these *vcrABC *from the first industrial production of chloroethenes. By contrast, our most conservative estimate for the MRCA of contemporary *Dehalococcoides *strains is 40,000 years ago (ranging as high as 3 Mya, Table 1), long before industrial civilization had a chance to influence the evolution of *Dehalococcoides *and their streamlined genomes specialized for organohalide respiration.

It is important to note that these molecular dating estimates use the available *vcrABC *sequences to predict the first horizontal acquisition of *vcrABC *by *Dehalococcoides*. This analysis is not meant to predict the age of genesis of the first vinyl chloride reductase. We did not detect partial homology with other *rdhA *that would suggest *vcrA *is a chimera resulting from a recent homologous recombination event. Moreover, the existence of an alternate vinyl chloride reductase from strain BAV1, BvcA [19], that shares deeply branching ancestry with VcrA on a tree of available RdhA [9], suggests that vinyl chloride reductases have existed for a considerable period of time, just not within strains of *Dehalococcoides *for which sequence data is currently available. In fact, naturally occurring vinyl chloride has been detected in soils [50], providing a plausible source of selective pressure to explain the existence of vinyl chloride reductases in nature prior to human pollution. However, we have not identified any candidate lineages as the possible progenitor of vinyl chloride reductases, and we have no way of knowing whether the primary substrate for the ancestral VcrA or BvcA was consistently vinyl chloride, leaving their ancestral history unclear.

The phylogenetic discord between integration modules and their attached *vcrABC *indicates that homologous recombination - or perhaps a more directed form of 'module swapping' - has recently occurred between *vcr-*GIs (Figure 3). This additional inter-element recombination may be independent of *ssrA*-specific integration, but it would still require horizontal transfer so that 2 or more *vcr-*GIs are collocated within the same cell. Multiple *vcr-*GI variants have not been detected in the same complete genome. However, we did detect a low-coverage variant in the KB-1 metagenome assembly with 3 corroborating reads that perfectly match a different *vcr-*GI integration module found in VS, WL, GT, and WBC-2 cultures, providing preliminary evidence of the physical collocation of two *vcr-*GIs within the KB-1 culture (Additional file 5 Figure S4).

### *ssrA*-GIs appear to be integrative and mobilizable elements

A subset of *Dehalococcoides rdhA*B were previously implicated in horizontal transfer [31,32], including the trichloroethene reductase gene, *tceAB *[32]. Although the selective conditions in chloroethene-contaminated environments favors maintenance of *tceAB *and *vcrABC*, the genes implicated in *tceAB *transfer [32] share no detectable homology with the *ssrA*-specific system described in detail here. We hypothesize that these *Dehalococcoides ssrA*-GIs behave as integrative and mobilizable elements ("IMEs") because they do not appear to encode conjugation, although they share many other features of the broadly defined class of integrative and conjugative elements ("ICEs") [33]. It may be possible that conjugation is encoded by a surprisingly minimal gene set within the integration modules [33], similar to the small (10.9 kbp) integrating and conjugating element 'pSAM2' of *Streptomyces ambofaciens*, which requires only a single gene, *traSA*, for inter-mycelial (conjugal) transfer [51]. *Dehalococcoides *core genes do include putative *pil *genes, the functions of which are unclear but may play a role in conjugation. Some strains of *Dehalococcoides *contain unambiguous prophages, providing an alternative hypothesis for the mechanism of *ssrA*-GI transfer, via illegitimate packaging of the excised *ssrA*-GI into a phage capsule. The length of *Dehalococcoides ssrA*-GIs is within the range of typical phage genomes. However, evidence for a complete prophage is not as ubiquitous among *Dehalococcoides *as the presence of *ssrA*-GIs, and there have been no descriptions to date of *Dehalococcoides *phage that also encode an *rdhA*, leaving the influence of phage on *rdhA *evolution unclear. Based on currently available evidence, we hypothesize that *Dehalococcoides ssrA*-GIs are mobilizable but not conjugating elements that sometimes mobilize adjacent tandem islands but in all cases rely on a host- or phage-encoded system for cell-cell transfer of a transient, presumably circular, intermediate.

*Dehalococcoides *also contains *comEA*, and it is unknown if *Dehalococcoides *is transiently competent for uptake of exogenous DNA. However, transfer via stochastic competence is an unsatisfying explanation, mainly because *Dehalococcoides ssrA*-GIs appear to lack genes encoding independent replication, and stable non-phage extrachromosomal elements have not been observed in *Dehalococcoides *[7-9].

Occasionally integrating and conjugating elements do have replicative forms [33], as in the case of rolling circle replication of pSAM2 in the donor cell [52]. Maphosa et al. recently described a field site in which there were 1 to 2 orders of magnitude more *vcrA *copies detected than copies of *tceA*, *bvcA*, or *Dehalococcoides *16S rRNA genes [53]. *vcrA *was also more abundant than *Dehalococcoides *16S rRNA genes in a dechlorinating bioreactor inoculated from the site [53], suggesting either (1) there exists a *vcr-*IME that can replicate independently or has integrated within an element that can replicate independently, or (2) they detected a non-*Dehalococcoides *population that also possesses *vcrA*, coexisting with a *Dehalococcoides *population.

It is important to note that, while a conspicuous and common feature, not all *Dehalococcoides ssrA*-GIs contain an integration module. We identified 15 *ssrA*-GIs without integration modules, containing a total of 38 *rdhA *as well as other genes. These might be 'cis-mobilizable elements' that encode neither integration nor transfer, but retain functional *attL*/*attR *sites [33] and are occasionally or constitutively mobilized with adjacent genomic islands through a process known as accretion [54]. In some cases these tandem *ssrA*-GIs may have been previously mobile but are now fixed in the chromosome. For example, there is a region immediately downstream of the direct repeats furthest from *ssrA *that is similarly dense in *rdhA *while also syntenic across *Dehalococcoides *strains, phylogenetically coherent with whole genome estimates, and devoid of *ssrA*-GI signatures (Additional file 6 Figure S5); suggesting this region was present in the MRCA of available *Dehalococcoides *[9]. Some or all of this region may have been acquired originally as an *ssrA*-GI, but deletion and amelioration has erased evidence of horizontal gene transfer.

### Likely Roles within *ssrA*-GI Integration Modules

The first identified *Dehalococcoides ssrA*-specific integrase gene (*dsiB*) (DhcVS_1292) was sequenced following the original identification and characterization of VcrA, and noted for its proximity to *vcrA *on the chromosome [5]. It is now clear that DhcVS_1292 is part of an integration module in an adjacent downstream *ssrA*-GI (GI 02 in VS, Figure 1), one of 16 *dsiB *homologs detected in *Dehalococcoides *genome sequences. The closest relative to *dsiB *in the public database is present on a fully-sequenced metagenomic fosmid from a deep (4000 m) ocean subsurface sample (EU016565, Figure 2), within an apparent integration module that also includes homologs to *dsiA*, *parB*, *mom*, and a putative tRNA embedded in *mom*, as well as an unambiguous *ssrA*-direct repeat at the homologous *attL *position embedded in *dsiB *(Additional file 7 Figure S6). This is especially intriguing in light of the recent sequencing of 32 novel *rdhA *amplified from various marine subsurface sediments [55], many of which appear phylogenetically within a major *rdhA *branch (Cluster I [9]) that is otherwise populated only by *rdhA *from *Dehalococcoides *or *Dehalogenimonas*. Given this indirect evidence and the large diversity of organohalogens detected in marine systems [56], it is tempting to speculate that *Dehalococcoides *plays a role in these settings. However, in the absence of direct observation of *Dehalococcoides*-like microorganisms in marine (subsurface) settings, this role remains unclear.

A more sensitive database search indicated that DsiB is a structurally similar homolog of CcrB, containing the serine-recombinase-catalytic domain at the N terminus, as well as similar motifs along its ~500 residue length (mean 22% ID, Figure 2A). CcrB specifically integrates/excises the so-called '*Staphylococcus *Cassette Chromosome' (SCC [57]) family of mobile elements that are a vector of antimicrobial resistance (among other phenotypes [58,59]), with major consequences for hospitals and the greater community [60-63]. CcrB was shown to have DNA-binding and recombination activity for *attS *of SCC [64], but SCC integration [57] and *attB*-specific excision both required the product of a smaller, co-transcribed serine recombinase gene, *ccrA*, that does not encode a DNA-binding domain [64]. Similarly, *Dehalococcoides *integration modules encode on a putative operon a second, smaller serine recombinase, DsiA, that also lacks a detectable DNA-binding motif. *Dehalococcoides ssrA*-GIs and SCC also share overlapping size ranges and specifically integrate at a non-tRNA, single-copy essential gene. We hypothesize that integration/excision of *Dehalococcoides ssrA*-GIs occurs in a homologous mechanism to SCC, via DsiB in concert with DsiA, with other integration module elements likely playing a role in regulation of integrase/excisionase activity or modification of the excised element to facilitate transfer or maintenance. Unfortunately, the mode of SCC transfer among *Staphylococcus *is unclear [65], and so does not provide additional clues regarding a likely transfer mechanism.

Interestingly, *dsiB *is always found overlapping *attL *at its 3' end. A stop codon occurs only upstream of the genomic island, even if that means overlapping substantially with an adjacent genomic island or *ssrA *itself. Complimentary overlap of *ssrA *with small open reading frames has been detected in some bacteria with ambiguous implications [66]. It seems unlikely in this instance that the 3' terminal ~70 bp of *ssrA *also encode a functional region of *dsiB *on its complementary strand. Accordingly, alignments of DsiB are divergent at this portion of their sequence, both in length and amino acid similarity. The majority of *dsiB *is upstream of *ssrA *or its direct repeat, and already comprises the expected length for homologs of *ccrB *(1600 bp). In addition to a trivial explanation in which *dsiB *undergoes low-efficiency translation that is variable at the C-terminus, it may be that *dsiB *is only fully functional when encoded on the circularized element, or alternatively when encoded on the chromosome downstream of an adjacent genomic island containing the requisite 3' gene fragment. In any case, the overlap of *dsiB *with *attP*/*attL *leaves the stop codon of *dsiB *unclear, and may have functional relevance or affect regulation of *dsiB*.

## Conclusions

Structural comparison of new (meta)genomic data, as well as targeted sequencing from unsequenced vinyl chloride respiring enrichment cultures, resulted in identification of 8 homologous mobile elements containing the vinyl chloride reductase operon, *vcrABC*. These *vcr-*GIs are a subset of mobile genetic elements in *Dehalococcoides *that specifically integrate at the single-copy gene, *ssrA*. A detailed comparison of these *ssrA*-GIs allowed identification of the precise position of insertion, the direct repeat created by the insertion event, as well as a conserved module of syntenic integration-associated genes that includes the likely *ssrA*-specific integrase, which we named *dsiB. ssrA*-GIs are most likely 'integrating and mobilizable elements' (IMEs) that do not encode their own mechanism of cellular transfer. Core (meta)genome phylogenetic analysis allowed an estimation of timing of divergence of *Dehalococcoides *strains, between 40,000 and 400,000 years ago, suggesting that the specialization by *Dehalococcoides *for respiration of organohalide compounds far precedes industrial synthesis by humans. By contrast, time estimates for the first horizontal acquisition of *vcrABC *sequences by *Dehalococcoides *are not confidently distinguishable from the first industrial synthesis of chloroethenes ~100 years ago. Vinyl chloride reductases may be ancient, nevertheless, and the donor(s) of recent genetic diversity to *Dehalococcoides *remain undetermined.

## Methods

### Identification of *Dehalococcoides* sequences in metagenome data

For ANAS and KB-1 enrichment cultures, complete genomes have not been assembled. However, identification of *Dehalococcoides *contigs was performed by comparison with closely-related complete genomes of strains 195 and CBDB1, respectively. For KB-1, gap closure was performed to create a closed draft genome of the dominant *Dehalococcoides *strain in the metagenome, including primer-walking of gap-spanning fosmid inserts, as well as gap-spanning PCR amplification from an aliquot of the originally-submitted KB-1 genomic DNA. For ANAS, structural genomic information comes from a single contig (contig 2014738012; 119815 bp) that contains a *Dehalococcoides ssrA *on one end and a series of tandem *ssrA*-GIs downstream (Figure 1). A second contig containing a *Dehalococcoides ssrA *was also detected (2014739670), but it contained no detectable *ssrA*-GIs. *Dehalococcoides *orthologs present in the ANAS dataset were detected using reciprocal best-hit BLASTp criteria described previously [8,9], treating the collection of all *Dehalococcoides *protein encoding genes in ANAS as if it were one genome. Although ANAS contains more than one *Dehalococcoides *strain, this approach had little effect on the number of orthologous groups considered 'core' between all *Dehalococcoides*, mainly because sequencing was sufficiently deep and because the *Dehalococcoides *strains in ANAS are closely-related to the Cornell lineage from which *ethenogenes *195 is the only completely-sequenced representative [67].

### DNA Source, Primer Design, Amplification Optimization

Enrichment cultures were derived from samples from geographically distinct contaminated sites (Additional file 8 Figure S7): WL from Western Louisiana [30]; EV from the Evanite site in Corvallis, Oregon [28]; PM from the Point Mugu Naval Weapon Facility, California [28]; and WBC-2 from the West Branch Canal Creek, Aberdeen Proving Ground, Maryland [29]. Culture, culture pellets, or genomic DNA were provided by L. Semprini (EV, PM) or E. Edwards (WL, WBC-2).

Nucleotide positions strongly conserved at *ssrA*, its direct repeat, and a few locations within integration modules were used for primer design (Additional file 2 Figure S2). Amplification was successful with a variety of combinations of predicted melting temperature and degeneracy. We described only the best-performing primer pairs, especially those that contributed to *vcr-*GI amplification and sequencing. All PCR amplifications from mixed cultures were performed using Phusion polymerase under standard conditions using 'HF' buffer. Primer pairs were optimized toward amplification of regions of *ssrA*-GIs in mixed genomic DNA template by DMSO and annealing temperature gradients. For most target amplicons the optimal DMSO concentration was at or near 4%, with optimal annealing temperature depending on the primer, and summarized in Additional file 2 Figure S2. In particular, a 20 bp forward primer beginning at *Dehalococcoides ssrA *position 8 (CGTGGTTTCGACAGGGAAGG - '*ssrA*_03F'), successfully amplified ~90% of all 4 novel *vcr-*GIs when paired with a reverse primer upstream of *vcrA *(GTTCCTGACCATGCCGTACC - 'vcrA_05R'). The resulting (8.3 - 10.5 kbp) amplicons were purified in agarose gel electrophoresis and sequenced directly by the Sanger method (MCLAB, ELIM) and primer walking. No single primer-pair was determined that could amplify a complete *ssrA*-GI in one reaction from *attL *to *attR*, as these would be reverse complements of one another and produce primer dimers. Instead, combinations of PCR reactions were amplified and sequenced separately, and their resulting sequence data was assembled *in silico *and verified manually. For example, forward primers targeting a conserved position in the integration module (TGGAGCGCCGCCGTNGG - 'REC_003eF') amplify a portion of the integration module and all of the genetic cargo (~7 kbp) when coupled with a reverse primer that targets the *ssrA*-direct repeat (TGGTGGAGACGGGGGAGGG - 'REC_001eR'). Two-fold or greater coverage and perfect agreement between different amplicons from the same sample was required in assembly. In some instances *ssrA*-GI-derived amplicons were cloned in *Escherichia coli *following agarose gel purification. Efficient ligation to a vector was achieved with Enzymatic Assembly [68] and pSMART-LC-Kan (AF532106; Lucigen Corp.) or by blunt ligation into the pJAZZ-OK linear vector (FJ160465; Lucigen). Transformation was achieved chemically in *E. coli *DH5*α *or electrically in *E. coli *BigEasy-TSA (Lucigen) cells, respectively.

### Core Genome and Genomic Island Phylogenies

The reciprocal BLASTp procedure for identifying orthologous groups among *Dehalococcoides *was also applied to *Dehalogenimonas lykanthroporepellens *BL-DC-9 in comparison to *Dehalococcoides *ANAS, KB-1, 195, DONNA2, CBDB1, BAV1, GT, and VS; resulting in 432 core orthologous groups that were also free of paralogs. Global alignments of each orthologous group were performed by Muscle (version 3.8.31) [69]. Single gene trees were generated using RAxMLHPC (version 7.0.3) [70] under the GTR + γ model [71] with *Dehalogenimonas lykanthroporepellens *BL-DC-9 constrained as the outgroup to prevent long-branch artifacts. The resulting trees were entered into Splitstree4 [72] and a consensus network was generated. The single gene global alignments were concatenated to generate a single large alignment for the 9 organisms. A core-genome phylogeny was generated using RAxMLHPC as described above, with 10 initial random starting tree iterations and 100 bootstrap replications. The tree with the highest likelihood is presented in Figure 4 and used for evolutionary analysis. Alignments for components of genomic islands were generated using Muscle and refined with hmmer (version 2.3.2) [73], then masked manually. Phylogenies were generated in RAxMLHPC under the GTR + γ model with 10 random starting trees and 100 bootstrap replications. In each case, the appropriate sequence was constrained as an outgroup. The trees with the best likelihood were identified and used for further age estimate calculations.

### Date Estimations

Estimates of the age of the *Dehalococcoides*/*Dehalogenimonas *divergence, the *Dehalococcoides *clade, as well as the various components of the genomic islands were determined under three different estimates for the rate of *Dehalococcoides *evolution. Two mutation rates from published values were used: one from a universal estimate of bacterial mutation rates in natural environments [74], and one from an empirical analysis of *E. coli *in lab cultures [75] (Additional file 3 Table S1). A third rate was based on a known divergence time of approximately 16 years between the separation of *Dehalococcoides ethenogenes *strain 195 ("strain 195") [6] from its mother culture - the "TCE/MeOH" culture (Prof. S. Zinder, pers. comm.) - in 1992, and the 2008 metagenome sequencing of the "DONNA2" enrichment culture. DONNA2 was also derived from the TCE/MeOH culture and maintained in parallel from strain 195 until its subsequent metagenome sequencing (R. E. Richardson, pers. comm., see DONNA2 Mutation Detection, below). Branch lengths between strain 195/DONNA2 were calculated from single-gene trees of the 387 core protein encoding genes, after excluding 45 trees that did not have strain 195 and DONNA2 as a monophyletic group, most likely resulting from frame-shift mutations. The mean branch length of the 387 protein encoding gene trees, the core-gene concatenated ML tree, as well as the splitstree [72] network average branch length were all approximately 3(10) ^-5^. For a minimum separation of 16 years, this corresponds to 2(10) ^-6 ^branch length per year of *Dehalococcoides *divergence. It is important to note that some unknown fraction of the observed mutations could have already existed within the mother culture prior to isolation of strain 195 if parents of the two contemporary strain variants coexisted at that time. Combined with the imposed pressures for rapid growth inherent to a laboratory culture, we expect that the *Dehalococcoides *mutation rates observed by this approach represent an unrealistic upper bound to what is likely to occur in *Dehalococcoides *in nature. This value is still useful, however, for creating lower bounds in molecular dating estimates that are compared with relatively recent events (e.g. human civilization, anthropogenic chloroethene pollution, etc.).

### DONNA2 *Dehalococcoides* Mutation Detection

Because the dominant bacterium in the DONNA2 culture was our target variant of strain 195, the DONNA2 metagenome data included a high coverage of this variant. A comparative assembly of the DONNA2 shotgun reads on the strain 195 genome allowed identification of reliable mutations between these two strains, using the Variant Ascertainment Algorithm (VAAL) under default settings [76]). The DONNA2 metagenome project has gone through successive rounds of sequencing, and the mutation analysis described here is based on the raw 454 GS FLX Titanium shotgun reads available on 06 November 2009, which were subsequently filtered by alignment to the genome sequence of *Dehalococcoides *strain 195. The resulting 455,062 *Dehalococcoides*-derived reads had a mean length of 365 ± 142 nucleotides, and %(G+C) of 48.8. Our version of VAAL did not produce assembly statistics, but a separate comparative assembly using Geneious Pro v5.4 (medium-sensitivity default parameters) successfully aligned 454,342 reads to the strain 195 genome, for a coverage of 115.2 ± 41.2. The consensus sequence of the comparative assembly produced by VAAL formed the basis for the subsequent strain-level mutation analysis. Gene annotations from strain 195 were mapped onto the DONNA2-variant genome sequence and the protein-encoding genes among these were extracted and included as a separate whole-genome collection in the genome-wide core gene phylogenetic analysis (above). The cumulative length of the protein encoding genes shared between 195 and DONNA2 was 1,301,665 bp; and among these genes we detected a total of 192 mutations, with adjacent SNPs considered part of a single mutation. Of these 192 mutations, 39% were deletions, 28% were insertions, 28% were transitions, and 4% were transversions. With respect to the predicted effects relative to the encoded protein in strain 195, 40% were frame-shift mutations, 40% were synonymous (probably no change), 19% were non-synonymous substitutions, and 1% were predicted to cause a truncation due to an early stop codon. It should be noted that frame-shift and truncation mutations would probably not directly affect our subsequent tree calculations because those genes would likely fail our orthology criteria (above) and thus would not be included in the set of "core" genes.

### Ka/*Ks *ratios

*K_a_*/*K_s _*ratios are an intrinsically pairwise calculation that was performed on a subset of the most different pairs of *vcrA *(full-length, and leader sequence only) using the 'kaks' function in the SeqinR package [44] of R [77]. *K_a_*/*K_s _*ratios were also calculated for all adjacent branches in a phylogenetic tree of the 8 sequences, using the Ka/Ks Calculation tool [45].

### Integration Module tRNA Secondary Structure

The putative tRNA sequence was originally detected by ARAGORN [78] and annotated previously in publicly available annotations of *Dehalococcoides *strains CBDB1 and VS. Secondary structure was predicted from the alignment of all 16 detected tRNAs in available *ssrA*-GI integration modules, submitted to the RNAalifold [79,80], Pfold [81], and PETfold [82] web servers for independent calculations. The resulting structures were compared manually, including a comparison to classical tRNA secondary structure for identification of the conserved "DCC" anti-codon within a 5 nt anti-codon loop (Additional file 1 Figure S1).

## Abbreviations

***vcrABC***: the vinyl chloride reductase operon; ***ssrA***: the tmRNA encoding gene; ***ssrA*-GI**: *ssrA*-specific genomic island; *vcr*-**GI**: a *vcrABC *-containing *ssrA*-specific genomic island, a subclass of *ssrA*-GI; **DsiB**: predicted *Dehalococcoides ssrA*-specific integrase; ***dsiB***: gene encoding DsiB; **MRCA**: most recent common ancestor; **ML**: Maximum Likelihood; **DMSO**: dimethyl sulfoxide; **CRISPR**: clustered regularly interspaced short palindromic repeats; **SCC**: Staphylococcus Cassette Chromosome; ***attL*/*attR***: the extreme left or right edge, respectively, of the genomic island that likely participates in site-specific recombination; **attB/attP**: the DNA motif on the bacterial chromosome or mobile element, respectively, that likely participates in site-specific recombination; **IME**: integrative and mobilizable elements; **ICE**: integrative and conjugative elements; **pSAM2**: an integrative and conjugative plasmid found in many *Streptomyces*; **EV, PM, WBC2, WL, ANAS, KB1, DONNA2***: Dehalococcoides *enrichment culture names; **CBDB1, GT, VS, BAV1**: *Dehalococcoides *strain names.

## Authors' contributions

PJM conceived of the experiments, carried out the molecular experiments, performed the comparative analyses, and drafted the manuscript. LAH maintained the WBC-2 and WL cultures, assisted with the molecular experiments, performed the molecular dating analyses and helped to draft the manuscript. EAE participated in the design of the study and helped to draft the manuscript. SH consulted on the molecular dating analyses and helped to draft the manuscript. AMS participated in the design of the study and helped to draft the manuscript. All authors read and approved the final manuscript.

## Description of additional data files

Additional data file 1 is a PDF format file containing the supplemental figures and associated legends. Additional data file 2 is a Microsoft excel (.xls) file containing tables of growth rates and rates of evolution, as well as other parameters and example calculations used in the molecular dating analyses.

## Supplementary Material

Additional file 1 **Figure S1: Alignment and Predicted Secondary Structure of Putative tRNA-gly**. These tRNA-gly are strongly conserved in 16 *Dehalococcoides ssrA*-GI integration modules. Bases are shaded according to the Vienna RNA conservation coloring schema in both the alignment (A) and secondary structure cartoon indicating the majority consensus with degeneracy (B). Secondary structure prediction was unanimous from three independent secondary structure prediction servers [80-82]. Free energy of the thermodynamic ensemble is -54.26 kcal/mol [80]. Substructure labels correspond to classical tRNA, including the apparent anti-codon 'DCC'. Click here for file

Additional file 2 **Figure S2: Primers Mapped onto an Alignment of 16 *ssrA *Integration Modules**. (A) Annotated alignment of the 16 integration modules discussed in this study. Individual sequences are shown as a thick black line, with gaps indicated by a thin horizontal line. Plot of average nucleotide identity (14 bp window) for all 16 sequences is shown along the top of the alignment. Three main target locations for primer design are indicated with downward-pointing black triangles, numbered beginning at *ssrA *(left). (B) Zoomed-in view of the alignment at the three target locations for primer binding. The 75% Consensus sequence is depressed slightly at the region targeted by primers, which are annotated along the top. Exact position of putative tRNA-gly is also shown. Click here for file

Additional file 3 **Figure S3: Phylogenetic Tree of *ssrA *Versus 16S rRNA gene**. The most likely of 100 bootstrap Maximum Likelihood trees with bootstrap support shown at nodes. Support not shown at nodes with poor or ambiguous support. (A) Phylogenetic tree of *ssrA*, the ~350 bp gene encoding tmRNA. (B) Similarly calculated tree based on the 16S rRNA gene (~1500 bp), reflected relative to typical tree orientation to emphasize topological similarity with (A). Other Chlorofiexi are included, with *Staphylococcus aureus *as an outgroup. Full name and accession number correspond to the following abbreviations: *Dehalococcoides *- Dhc; CBDB1 - Dhc CBDB1 NC_007356; GT - Dhc GT NC_013890; BAV1 - Dhc BAV1 NC_009455; 195 - Dhc *ethenogenes *195 NC_002936; VS - Dhc VS NC_013552; Deha lyk - *Dehalogenimonas lykanthroporepellens *BL-DC-9 NC_014314; Staph aur - *Staphylococcus aureus *NC_002952; Rose cast - Ro-*seiflexus castenholzii *DSM 13941 NC_009767; Rose RS-1 - *Roseiflexus *sp. RS-1 NC_009523; Chlo aur - *Chloroflexus aurantiacus *J-10-fl NC_010175; Chlo agg - *Chloroflexus aggregans *DSM 9485 NC_011831. Click here for file

Additional file 4 **Table S1: Parameters and example calculations utilized in divergence age estimates**. **(Top table) **Summary of age estimates for *Dehalococcoides*-related genetic divergence utilizing four different models for rate of evolution: (1) estimated universal bacterial rate of evolution in nature [74], (2) *in vitro E. coli *empirically derived rate of evolution [75], (3) empirical *Dehalococcoides *rate based on observed mutations in the whole genomes of strain 195 and its resequenced variant in the DONNA2 sister culture (see Methods), and (4) the 16S rRNA gene clock model. For ages based on the first two rates of evolution, we further considered six different values for doubling time that span a range relevant to *Dehalococcoides*, including four published values for *Dehalococcoides *growth in laboratory culture [4,6,88,96], other anaerobic bacterial growth rates [47], and values derived from environmental anaerobic systems [48,49], as well as one arbitrarily large value (130 days) intended to represent general substrate-limited conditions. The left two columns indicate the divergence being considered and the tree calculation method, respectively. Ages are presented in units of 1 million years. **(Middle Two Tables) **Referenced summary of growth rates utilized for the age estimate calculations. **(Bottom Table) **Sample calculation for length of time to a single mutation, given rates of evolution taken from literature and the averaged *Dehalococcoides *growth rate. Click here for file

Additional file 5 **Figure S4: KB-1 variant at *vcr*-GI module transition**. (A) Cartoon representation of the *vcr-*GI observed in all 8 versions, as shown in Figure 3. (B) Alignment of the region at the transition between integration and *vcrABC *cargo modules, including reads in the KB-1 metagenome dataset that disagree with the main consensus at this location. All 3 of these variant reads are perfectly identical to the VS, GT, WL, and WBC-2 *vcr-*GIs at this position. Click here for file

Additional file 6 **Figure S5: Genetic Map of Putative Fixed *rdhA *Region Downstream of Direct Repeats**. (Top) Genetic map output from a Mauve alignment of the portion of High Plasticity Region 2 (HPR2) downstream of any *ssrA *direct repeats in the *Dehalococcoides *genomes. Each sequence was first aligned at tRNA-Ala-3 previously defining the boundary of HPR2 closest to the Ori [9], with local collinear blocks (LCBs) indicating large collinear homologous region that are free from rearrangements, but not necessarily indels. Large gaps were manually inserted such that vertical positions also containing the identity graph indicate aligned positions within the LCB. The darker grey LCB is the putative 'fixed' region of HPR2 downstream of any *ssrA *direct repeats. The lighter grey LCB is a portion of the *Dehalococcoides *core genome that surrounds the Ori. Annotated genes are shown beneath each LCB, with genes on the forward and reverse strands drawn as rectangles above or below the midline, respectively. *rdhA *are shaded red for emphasis. Scale bar shown in top left corner. Note that two different contigs from the ANAS genome are included. (Bottom) Phylogenetic trees of three semi-core (missing strain BAV1) *rdhA *that share a syntenic neighborhood within the putative fixed region. Each orthologous *rdhA *group recapitulates the topology and approximate genetic distances of the whole-genome tree (Figure 4). HPR2 was deleted in strain BAV1 [9], save for a ~600 bp *rdhA *fragment (DehaBAV1_1302) that is the basis for the tree on the right-hand side. Click here for file

Additional file 7 **Figure S6: Genetic Map of a *dsiB*-Containing Deep-Sea Environmental Fosmid**. The fosmid, EU016565, contains the most similar non-*Dehalococcoides *integration module(s) detected in the public database. EU016565 is part of an environmental shotgun sequencing dataset of genomic DNA obtained from a 4000 m sub-seafloor sediment [87]. Two partial *Dehalococcoides ssrA *integration modules are detectable, one of which contains an *ssrA *direct repeat at the expected location within a *dsiB *homolog. It also contains 4 of the 6 protein encoding genes typically found in integration modules as well as the putative tRNA embedded within *mom *homolog. The reverse-complement of EU016565 is displayed for consistent orientation with other figures. Light grey, dark grey, and black indicate protein encoding genes for which the annotation is hypothetical, identifiable, or part of the integration module, respectively. Click here for file

Additional file 8 **Figure S7: Geographic locations of *Dehalococcoides *strains and cultures mentioned in this article**. The underlying map was created using Google Earth. Labels have a dark red border if they are cultures/strains for which high throughput sequencing data is available and vinyl chloride respiration is reported. Blue borders indicate the vinyl chloride respiring cultures for which genomic island data was obtained during this study. White stars indicate cultures/strains for which no high throughput sequencing data was available at the time of this publication. The origin of the *Dehalococcoides *isolate FL2 [88] and the *Dehalococcoides *enrichment culture 'Pinellas' [89] are also shown. The following isolated bacterial strains were discussed in the manuscript: *Dehalococcoides *ethenogenes 195 - Ithaca Wastewater Treatment Plant, Ithaca, NY, USA [6,90]; CBDB1 - Saale River, Jena, Germany [91-93]; BAV1 - Bachman Road Site, Oscada, MI, USA [94]; VS - Contaminated Site, Victoria, Texas, USA [95]; GT - Hydrite Chemical Co., Cottage Grove, WI, USA [17]; *Dehalogenimonas lykanthroporepellens *BL-DC-9 [46]. The following *Dehalococcoides *enrichments were discussed. An asterisk indicates that no high-throughput sequence data is currently available: KB-1 - Southern Ontario, Canada [25]; ANAS - Alameda Naval Air Station, CA, USA [27] *PM - Point Mugu Naval Weapon Facility, CA, USA [28]; *EV - Evanite contaminated site, Corvallis, Oregon, USA [28]; *WBC-2 - West Branch Canal Creek, Aberdeen Proving Ground, MD [29] *WL - contaminated site, Western Louisiana, USA [30]. Click here for file
