# Supplementary material for: Site-Specific Mobilization of Vinyl Chloride Respiration Islands by a Mechanism Common in Dehalococcoides
Source: BMC Genomics. 2011 Jun 2;12:287. doi: 10.1186/1471-2164-12-287 (PMC3146451; doi:10.1186/1471-2164-12-287)
Supplement: Additional file 2 — Figure S2: Primers Mapped onto an Alignment of 16 ssrA Integration Modules. (A) Annotated alignment of the 16 integration modules discussed in this study. Individual sequences are shown as a thick black line, with gaps indicated by a thin horizontal line. Plot of average nucleotide identity (14 bp window) for all 16 sequences is shown along the top of the alignment. Three main target locations for primer design are indicated with downward-pointing black triangles, numbered beginning at ssrA (left). (B) Zoomed-in view of the alignment at the three target locations for primer binding. The 75% Consensus sequence is depressed slightly at the region targeted by primers, which are annotated along the top. Exact position of putative tRNA-gly is also shown. [file 1471-2164-12-287-S2.PDF]

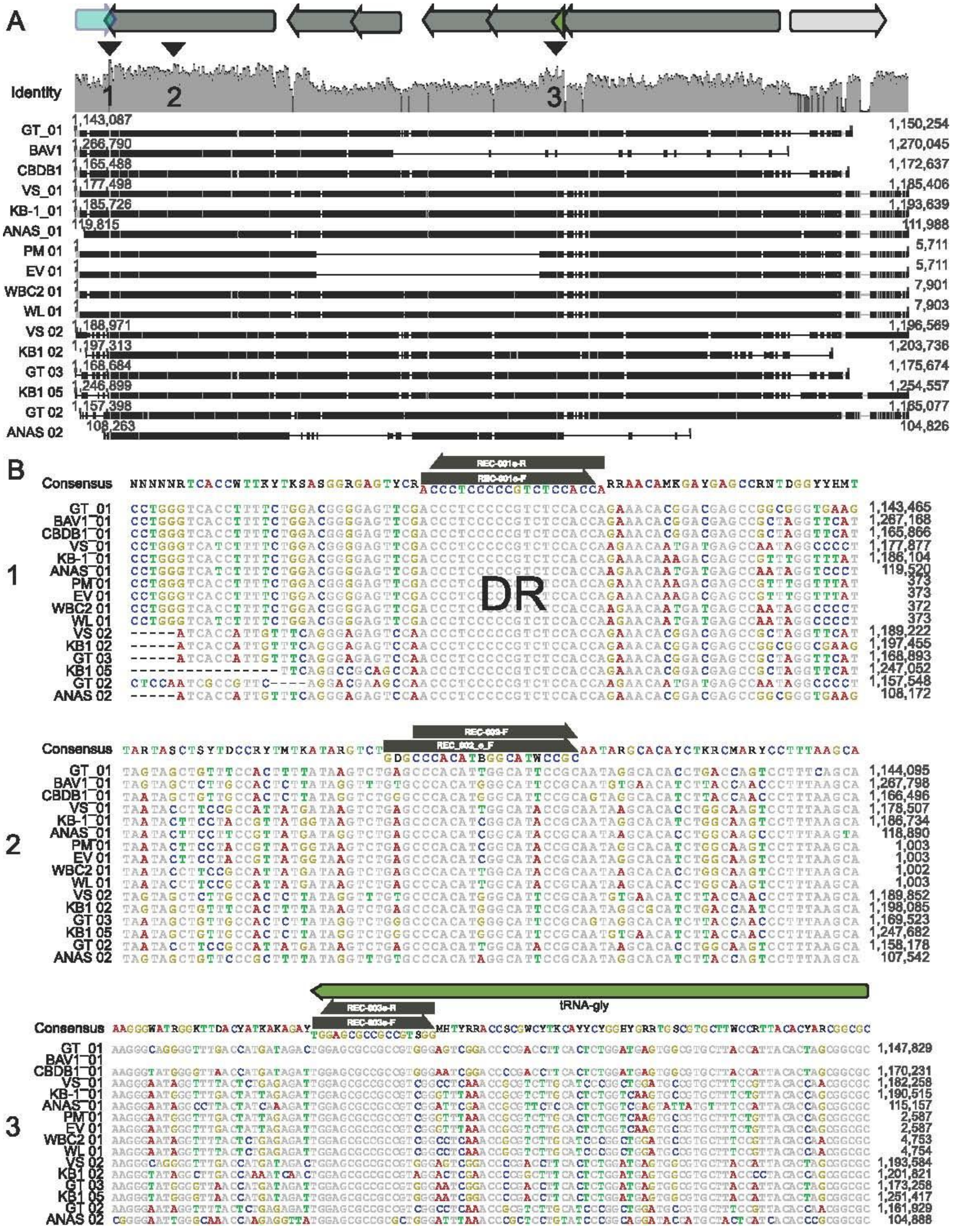

Figure S2: Primers Mapped onto an Alignment of 16 ssrA Integration Modules. (A) Annotated alignment of the 16 integration modules discussed in this study. Individual sequences are shown as a thick black line, with gaps indicated by a thin horizontal line. Plot of average nucleotide identity (14 bp window) for all 16 sequences is shown along the top of the alignment. Three main target locations for primer design are indicated with downward-pointing black triangles, numbered beginning at ssrA (left). (B) Zoomed-in view of the alignment at the three target locations for primer binding. The 75% Consensus sequence is depressed slightly at the region targeted by primers, which are annotated along the top. Exact position of putative tRNA-gly is also shown.
