# Supplementary material for: Site-Specific Mobilization of Vinyl Chloride Respiration Islands by a Mechanism Common in Dehalococcoides
Source: BMC Genomics. 2011 Jun 2;12:287. doi: 10.1186/1471-2164-12-287 (PMC3146451; doi:10.1186/1471-2164-12-287)
Supplement: Additional file 3 — Figure S3: Phylogenetic Tree of ssrA Versus 16S rRNA gene. The most likely of 100 bootstrap Maximum Likelihood trees with bootstrap support shown at nodes. Support not shown at nodes with poor or ambiguous support. (A) Phylogenetic tree of ssrA, the ~350 bp gene encoding tmRNA. (B) Similarly calculated tree based on the 16S rRNA gene (~1500 bp), reflected relative to typical tree orientation to emphasize topological similarity with (A). Other Chlorofiexi are included, with Staphylococcus aureus as an outgroup. Full name and accession number correspond to the following abbreviations: Dehalococcoides - Dhc; CBDB1 - Dhc CBDB1 NC_007356; GT - Dhc GT NC_013890; BAV1 - Dhc BAV1 NC_009455; 195 - Dhc ethenogenes 195 NC_002936; VS - Dhc VS NC_013552; Deha lyk - Dehalogenimonas lykanthroporepellens BL-DC-9 NC_014314; Staph aur - Staphylococcus aureus NC_002952; Rose cast - Ro-seiflexus castenholzii DSM 13941 NC_009767; Rose RS-1 - Roseiflexus sp. RS-1 NC_009523; Chlo aur - Chloroflexus aurantiacus J-10-fl NC_010175; Chlo agg - Chloroflexus aggregans DSM 9485 NC_011831. [file 1471-2164-12-287-S3.PDF]

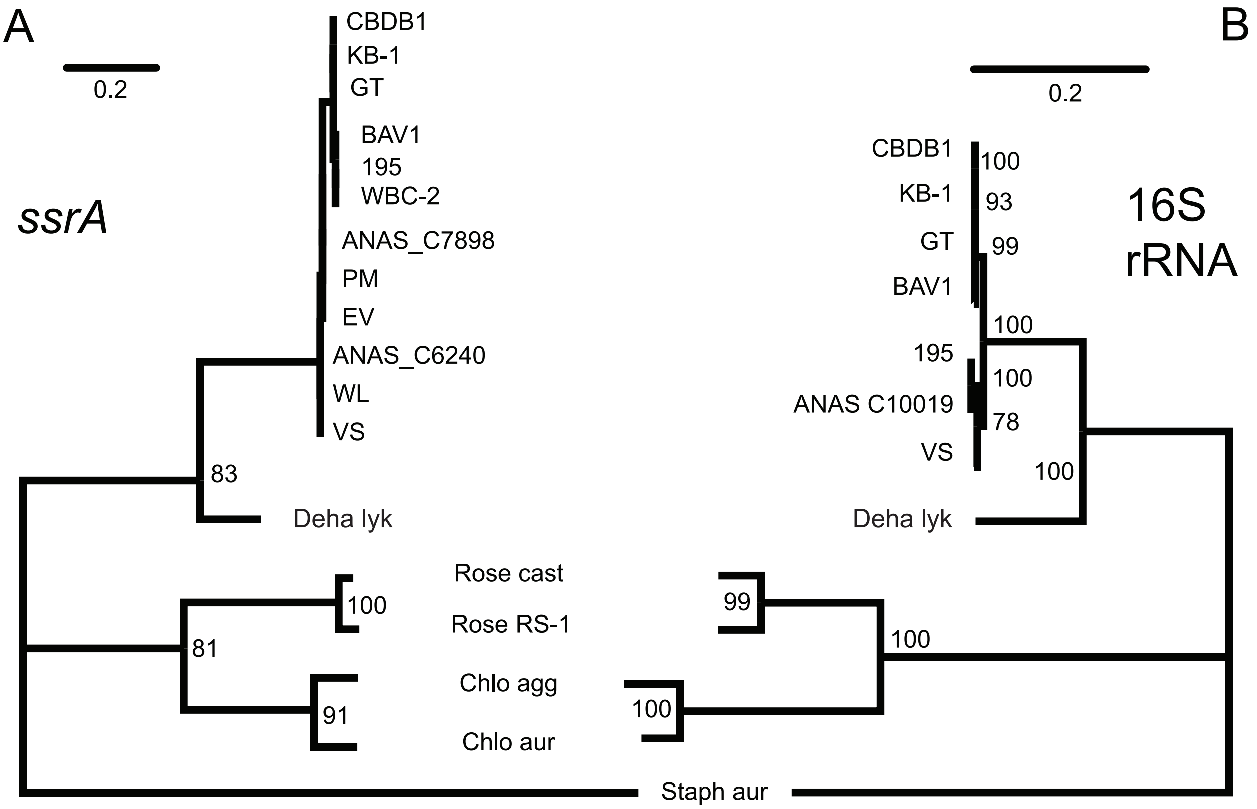

Additional file 1, Figure S3: Phylogenetic Tree of *ssrA* Versus 16S rRNA gene. The most likely of 100 bootstrap Maximum Likelihood trees with bootstrap support shown at nodes. Support not shown at nodes with poor or ambiguous support. (A) Phylogenetic tree of *ssrA*, the ∼350 bp gene encoding tmRNA. (B) Similarly calculated tree based on the 16S rRNA gene (∼ 1500 bp), reflected relative to typical tree orientation to emphasize topological similarity with (A). Other Chloroflexi are included, with *Staphylococcus aureus* as an outgroup. Full name and accession number correspond to the following abbreviations: *Dehalococcoides* - Dhc; CBDB1 - Dhc CBDB1 NC_007356; GT - Dhc GT NC_013890; BAV1 - Dhc BAV1 NC_009455; 195 - Dhc *ethenogenes* 195 NC_002936; VS - Dhc VS NC_013552; Deha lyk - *Dehalogenimonas lykanthroporepellens* BL-DC-9 NC_014314; Staph aur - *Staphylococcus aureus* NC_002952; Rose cast - *Roseiflexus castenholzii* DSM 13941 NC_009767; Rose RS-1 - *Roseiflexus* sp. RS-1 NC_009523; Chlo aur - *Chloroflexus aurantiacus* J-10-fl NC_010175; Chlo agg - *Chloroflexus aggregans* DSM 9485 NC_011831
