# Supplementary material for: Site-Specific Mobilization of Vinyl Chloride Respiration Islands by a Mechanism Common in Dehalococcoides
Source: BMC Genomics. 2011 Jun 2;12:287. doi: 10.1186/1471-2164-12-287 (PMC3146451; doi:10.1186/1471-2164-12-287)
Supplement: Additional file 5 — Figure S4: KB-1 variant at vcr-GI module transition. (A) Cartoon representation of the vcr-GI observed in all 8 versions, as shown in Figure 3. (B) Alignment of the region at the transition between integration and vcrABC cargo modules, including reads in the KB-1 metagenome dataset that disagree with the main consensus at this location. All 3 of these variant reads are perfectly identical to the VS, GT, WL, and WBC-2 vcr-GIs at this position. [file 1471-2164-12-287-S5.PDF]

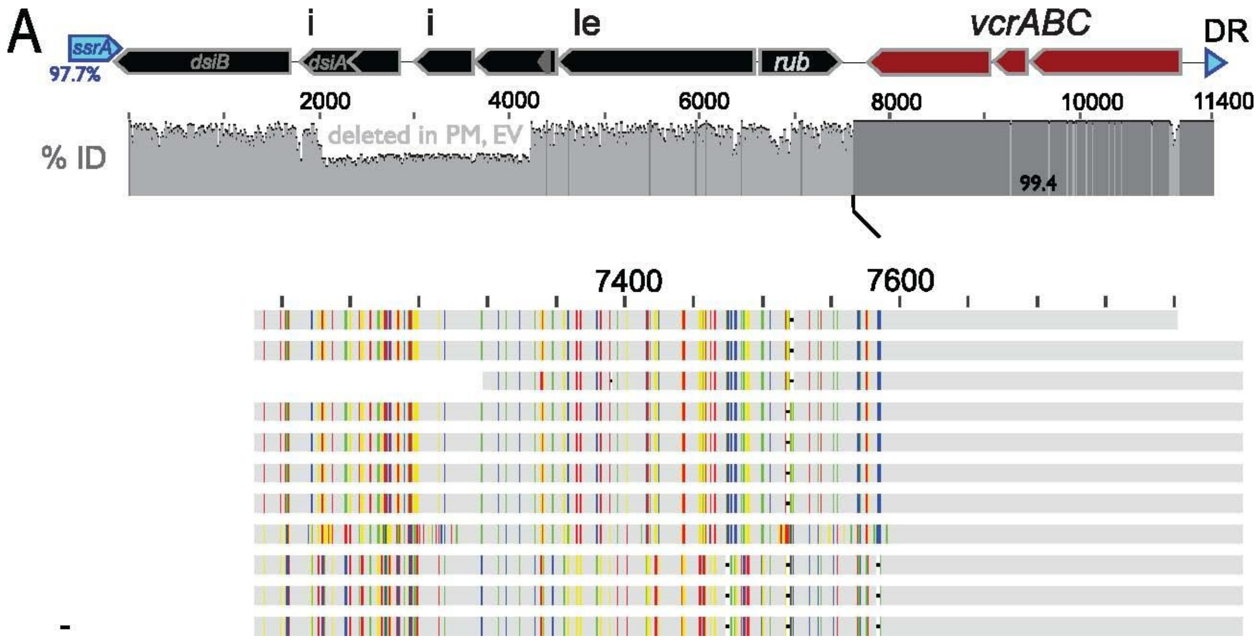

Figure S4: KB-1 variant at vcr-GI module transition. (A) Cartoon representation of the vcr-GI observed in all 8 versions, as shown in Figure 3. (B) Alignment of the region at the transition between integration and vcrABC cargo modules, including reads in the KB-1 metagenome dataset that disagree with the main consensus at this location. All 3 of these variant reads are perfectly identical to the VS, GT, WL, and WBC-2 vcr-GIs at this position.
