# Supplementary material for: Site-Specific Mobilization of Vinyl Chloride Respiration Islands by a Mechanism Common in Dehalococcoides
Source: BMC Genomics. 2011 Jun 2;12:287. doi: 10.1186/1471-2164-12-287 (PMC3146451; doi:10.1186/1471-2164-12-287)
Supplement: Additional file 6 — Figure S5: Genetic Map of Putative Fixed rdhA Region Downstream of Direct Repeats. (Top) Genetic map output from a Mauve alignment of the portion of High Plasticity Region 2 (HPR2) downstream of any ssrA direct repeats in the Dehalococcoides genomes. Each sequence was first aligned at tRNA-Ala-3 previously defining the boundary of HPR2 closest to the Ori [9], with local collinear blocks (LCBs) indicating large collinear homologous region that are free from rearrangements, but not necessarily indels. Large gaps were manually inserted such that vertical positions also containing the identity graph indicate aligned positions within the LCB. The darker grey LCB is the putative 'fixed' region of HPR2 downstream of any ssrA direct repeats. The lighter grey LCB is a portion of the Dehalococcoides core genome that surrounds the Ori. Annotated genes are shown beneath each LCB, with genes on the forward and reverse strands drawn as rectangles above or below the midline, respectively. rdhA are shaded red for emphasis. Scale bar shown in top left corner. Note that two different contigs from the ANAS genome are included. (Bottom) Phylogenetic trees of three semi-core (missing strain BAV1) rdhA that share a syntenic neighborhood within the putative fixed region. Each orthologous rdhA group recapitulates the topology and approximate genetic distances of the whole-genome tree (Figure 4). HPR2 was deleted in strain BAV1 [9], save for a ~600 bp rdhA fragment (DehaBAV1_1302) that is the basis for the tree on the right-hand side. [file 1471-2164-12-287-S6.PDF]

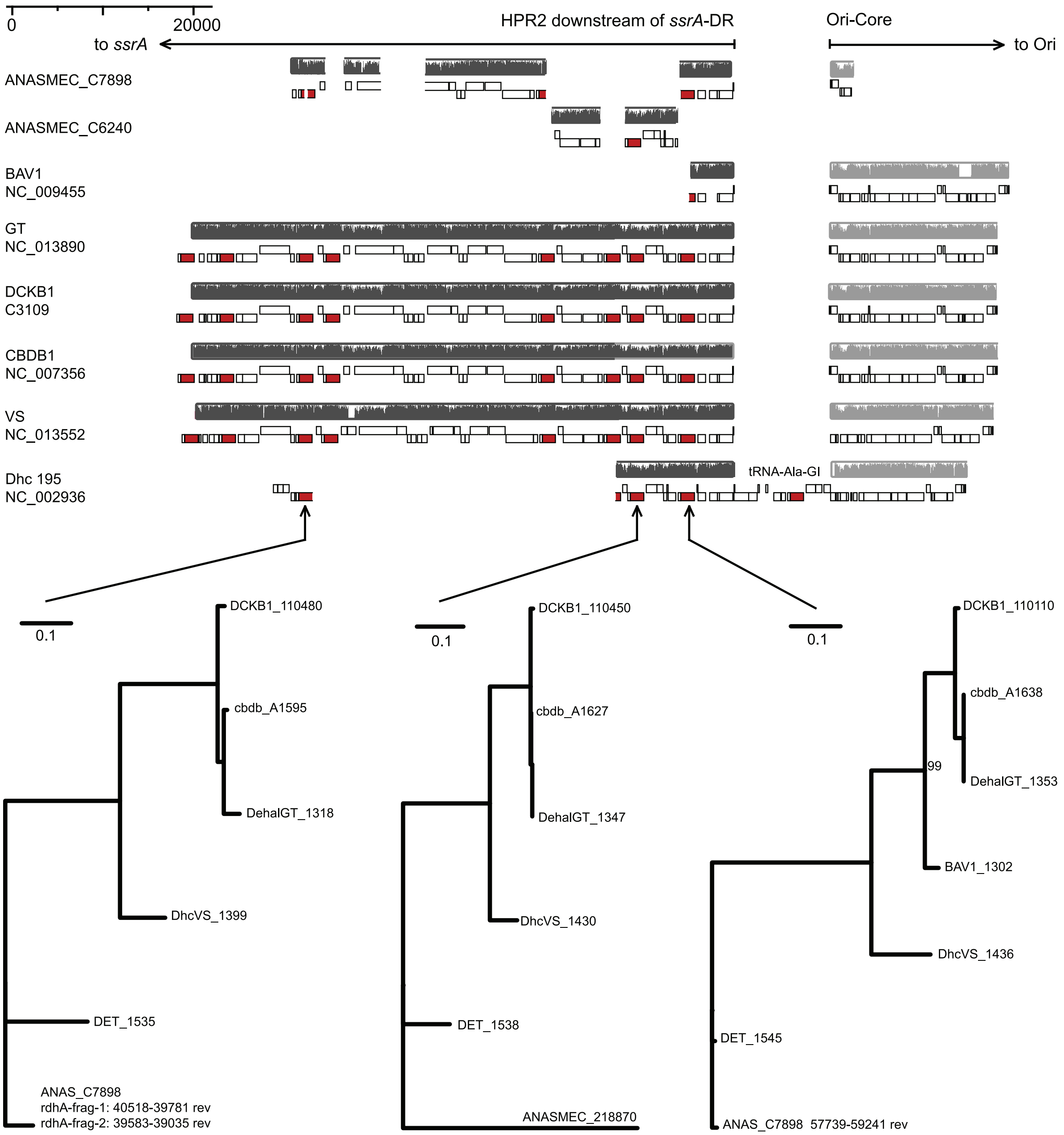

Additional file 1, Figure S5: Genetic Map of Putative Fixed *rdhA* Region Downstream of Direct Repeats. (Top) Genetic map output from a Mauve alignment of the portion of High Plasticity Region 2 (HPR2) downstream of any *ssrA* direct repeats in the *Dehalococcoides* genomes. Each sequence was first aligned at tRNA-Ala-3 previously defining the boundary of HPR2 closest to the Ori [9], with local collinear blocks (LCBs) indicating large collinear homologous region that are free from rearrangements, but not necessarily indels. Large gaps were manually inserted such that vertical positions also containing the identity graph indicate aligned positions within the LCB. The darker grey LCB is the putative 'fixed' region of HPR2 downstream of any *ssrA* direct repeats. The lighter grey LCB is a portion of the *Dehalococcoides* core genome that surrounds the Ori. Annotated genes are shown beneath each LCB, with genes on the forward and reverse strands drawn as rectangles above or below the midline, respectively. *rdhA* are shaded red for emphasis. Scale bar shown in top left corner. Note that two different contigs from the ANAS genome are included. (Bottom) Phylogenetic trees of three semi-core (missing strain BAV1) *rdhA* that share a syntenic neighborhood within the putative fixed region. Each orthologous *rdhA* group recapitulates the topology and approximate genetic distances of the whole-genome tree (Figure 4). HPR2 was deleted in strain BAV1 [9], save for a ∼600 bp *rdhA* fragment (DehaBAV1_1302) that is the basis for the tree on the right-hand side.
