# Supplementary material for: Site-Specific Mobilization of Vinyl Chloride Respiration Islands by a Mechanism Common in Dehalococcoides
Source: BMC Genomics. 2011 Jun 2;12:287. doi: 10.1186/1471-2164-12-287 (PMC3146451; doi:10.1186/1471-2164-12-287)
Supplement: Additional file 7 — Figure S6: Genetic Map of a dsiB-Containing Deep-Sea Environmental Fosmid. The fosmid, EU016565, contains the most similar non-Dehalococcoides integration module(s) detected in the public database. EU016565 is part of an environmental shotgun sequencing dataset of genomic DNA obtained from a 4000 m sub-seafloor sediment [87]. Two partial Dehalococcoides ssrA integration modules are detectable, one of which contains an ssrA direct repeat at the expected location within a dsiB homolog. It also contains 4 of the 6 protein encoding genes typically found in integration modules as well as the putative tRNA embedded within mom homolog. The reverse-complement of EU016565 is displayed for consistent orientation with other figures. Light grey, dark grey, and black indicate protein encoding genes for which the annotation is hypothetical, identifiable, or part of the integration module, respectively. [file 1471-2164-12-287-S7.PDF]

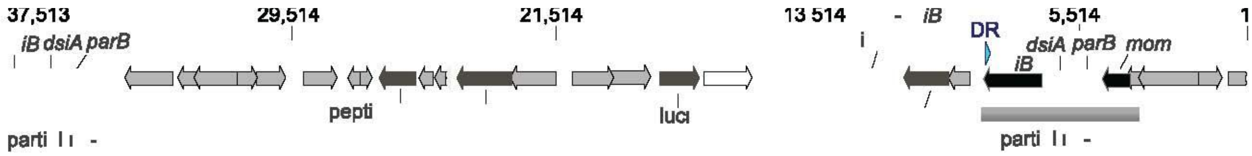

Figure S6: Genetic Map of a dsiB-Containing Deep-Sea Environmental Fosmid. The fosmid, EU016565, contains the most similar non-Dehalococcoides integration module(s) detected in the public database. EU016565 is part of an environmental shotgun sequencing dataset of genomic DNA obtained from a 4000 m sub-seafloor sediment [87]. Two partial Dehalococcoides ssrA integration modules are detectable, one of which contains an ssrA direct repeat at the expected location within a dsiB homolog. It also contains 4 of the 6 protein encoding genes typically found in integration modules as well as the putative tRNA embedded within mom homolog. The reverse-complement of EU016565 is displayed for consistent orientation with other figures. Light grey, dark grey, and black indicate protein encoding genes for which the annotation is hypothetical, identifiable, or part of the integration module, respectively.
