# Supplementary material for: Site-Specific Mobilization of Vinyl Chloride Respiration Islands by a Mechanism Common in Dehalococcoides
Source: BMC Genomics. 2011 Jun 2;12:287. doi: 10.1186/1471-2164-12-287 (PMC3146451; doi:10.1186/1471-2164-12-287)
Supplement: Additional file 8 — Figure S7: Geographic locations of Dehalococcoides strains and cultures mentioned in this article. The underlying map was created using Google Earth. Labels have a dark red border if they are cultures/strains for which high throughput sequencing data is available and vinyl chloride respiration is reported. Blue borders indicate the vinyl chloride respiring cultures for which genomic island data was obtained during this study. White stars indicate cultures/strains for which no high throughput sequencing data was available at the time of this publication. The origin of the Dehalococcoides isolate FL2 [88] and the Dehalococcoides enrichment culture 'Pinellas' [89] are also shown. The following isolated bacterial strains were discussed in the manuscript: Dehalococcoides ethenogenes 195 - Ithaca Wastewater Treatment Plant, Ithaca, NY, USA [6,90]; CBDB1 - Saale River, Jena, Germany [91-93]; BAV1 - Bachman Road Site, Oscada, MI, USA [94]; VS - Contaminated Site, Victoria, Texas, USA [95]; GT - Hydrite Chemical Co., Cottage Grove, WI, USA [17]; Dehalogenimonas lykanthroporepellens BL-DC-9 [46]. The following Dehalococcoides enrichments were discussed. An asterisk indicates that no high-throughput sequence data is currently available: KB-1 - Southern Ontario, Canada [25]; ANAS - Alameda Naval Air Station, CA, USA [27] *PM - Point Mugu Naval Weapon Facility, CA, USA [28]; *EV - Evanite contaminated site, Corvallis, Oregon, USA [28]; *WBC-2 - West Branch Canal Creek, Aberdeen Proving Ground, MD [29] *WL - contaminated site, Western Louisiana, USA [30]. [file 1471-2164-12-287-S8.PDF]

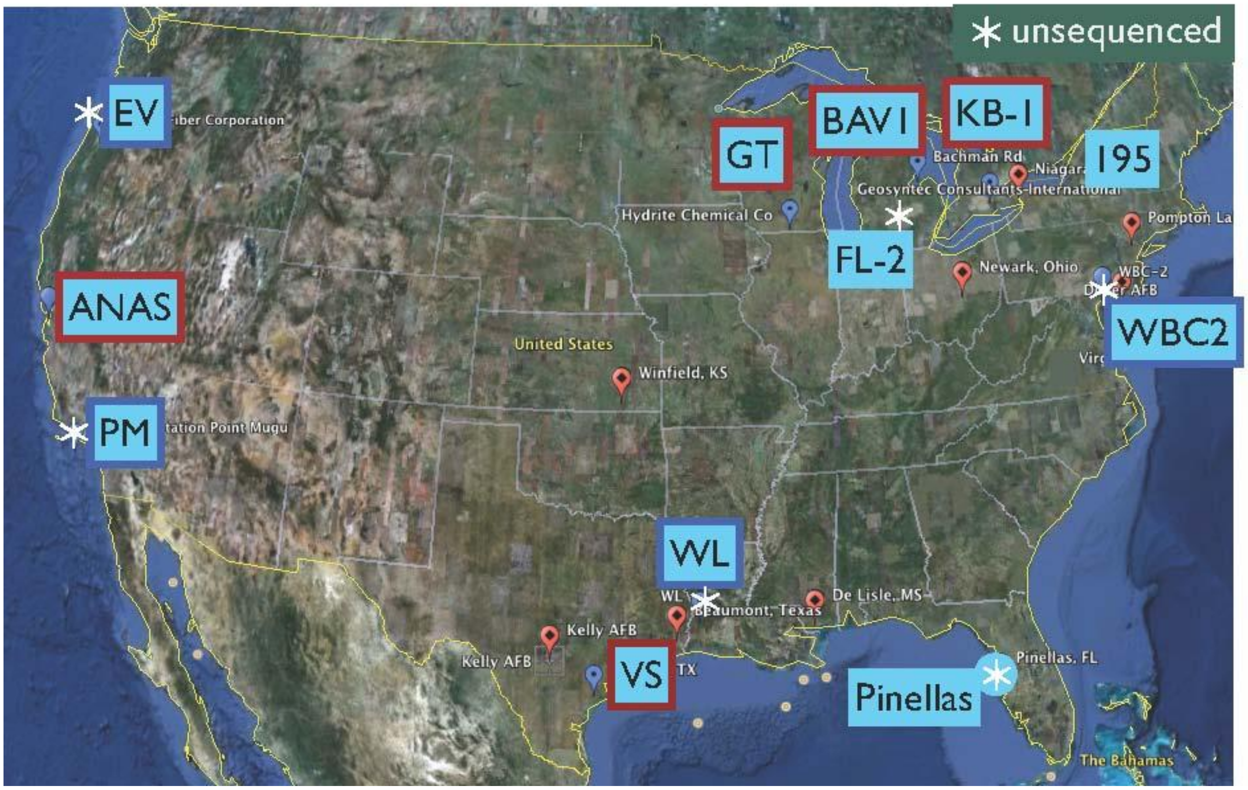

Figure S7: Geographic locations of Dehalococcoides strains and cultures mentioned in this article. The underlying map was created using Google Earth. Labels have a dark red border if they are cultures/strains for which high throughput sequencing data is available and vinyl chloride respiration is reported. Blue borders indicate the vinyl chloride respiring cultures for which genomic island data was obtained during this study. White stars indicate cultures/strains for which no high throughput sequencing data was available at the time of this publication. The origin of the Dehalococcoides isolate FL2 [88] and the Dehalococcoides enrichment culture 'Pinellas' [89] are also shown. The following isolated bacterial strains were discussed in the manuscript: Dehalococcoides ethenogenes 195 -Ithaca Wastewater Treatment Plant, Ithaca, NY, USA [6,90]; CBDB1 -Saale River, Jena, Germany [91–93]; BAV1 -Bachman Road Site, Oscada, MI, USA [94]; VS -Contaminated Site, Victoria, Texas, USA [95]; GT -Hydrite Chemical Co., Cottage Grove, WI, USA [17]; Dehalogenimonas lykanthroporepellens BL-DC-9 [46]. The following Dehalococcoides enrichments were discussed. An asterisk indicates that no high-throughput sequence data is currently available: KB-1 -Southern Ontario, Canada [25]; ANAS -Alameda Naval Air Station, CA, USA [27] *PM -Point Mugu Naval Weapon Facility, CA, USA [28]; *EV -Evanite contaminated site, Corvallis, Oregon, USA [28]; *WBC-2 -West Branch Canal Creek, Aberdeen Proving Ground, MD [29] *WL -contaminated site, Western Louisiana, USA [30]
