# Supplementary material for: Site-Specific Mobilization of Vinyl Chloride Respiration Islands by a Mechanism Common in Dehalococcoides
Source: BMC Genomics. 2011 Jun 2;12:287. doi: 10.1186/1471-2164-12-287 (PMC3146451; doi:10.1186/1471-2164-12-287)
Supplement: Additional file 1 — Figure S1: Alignment and Predicted Secondary Structure of Putative tRNA-gly. These tRNA-gly are strongly conserved in 16 Dehalococcoides ssrA-GI integration modules. Bases are shaded according to the Vienna RNA conservation coloring schema in both the alignment (A) and secondary structure cartoon indicating the majority consensus with degeneracy (B). Secondary structure prediction was unanimous from three independent secondary structure prediction servers [80-82]. Free energy of the thermodynamic ensemble is -54.26 kcal/mol [80]. Substructure labels correspond to classical tRNA, including the apparent anti-codon 'DCC'. [file 1471-2164-12-287-S1.PDF]

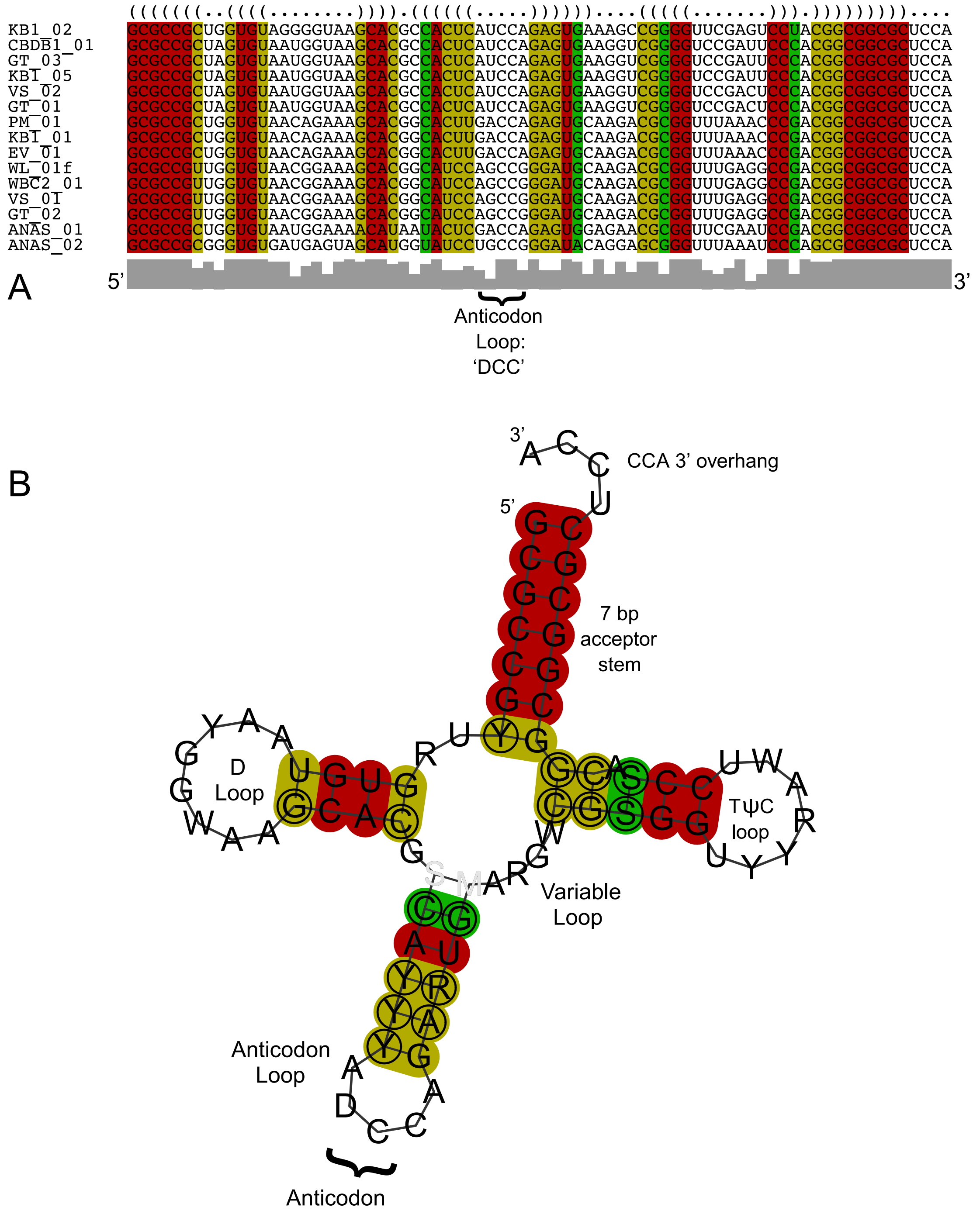

Additional file 1, Figure S1: Alignment and Predicted Secondary Structure of Putative tRNA-gly. These tRNA-gly are strongly conserved in 16 *Dehalococcoides ssrA*-GI integration modules. Bases are shaded according to the Vienna RNA conservation coloring schema in both the alignment **(A)** and secondary structure cartoon indicating the majority consensus with degeneracy **(B)**. Secondary structure prediction was unanimous from three independent secondary structure prediction servers [80–82]. Free energy of the thermodynamic ensemble is -54.26 kcal/mol [80]. Substructure labels correspond to classical tRNA, including the apparent anti-codon 'DCC'.
